# Effect of Manual Wheelchair Type on Mobility Performance, Cardiorespiratory Responses, and Perceived Exertion

**DOI:** 10.1155/2022/5554571

**Published:** 2022-06-11

**Authors:** Guilherme da Silva Bertolaccini, Frode Eika Sandnes, Fausto Orsi Medola, Terje Gjøvaag

**Affiliations:** ^1^Faculty of Technology, Art and Design, Oslo Metropolitan University, Oslo, Norway; ^2^School of Architecture, Arts and Communication, State University of Sao Paulo (UNESP), Bauru, Brazil; ^3^Faculty of Health Sciences, Oslo Metropolitan University, Oslo, Norway

## Abstract

This study is aimed at comparing the design and configuration of the most commonly used manual wheelchair models through cardiorespiratory responses, perceived exertion, and mobility performance using two different manual wheelchairs, during mobility tasks. A within-group 2 × 3 × 2 controlled experiment was designed with three independent and four dependent variables. The independent variables included wheelchairs, with the levels active wheelchair with a rigid frame and passive wheelchair with foldable frame; conditions with the levels straight line, slalom, and agility; and speed with levels comfortable and fast. Dependent variables included oxygen uptake (VO_2_), distance travelled, speed, and perceived exertion. Results show that the active wheelchair yielded more beneficial characteristics although only the effect of wheelchair type on VO_2_ efficiency (oxygen uptake per meter travelled) was statistically significant with a large effect size (*F*(1, 14) = 118.298, *p* < 0.001, *η*^2^ = 0.541). The better VO_2_ efficiency was achieved with the active wheelchair under all tested conditions. The effect of wheelchair type on Borg scores was also statistically significant, although with a small effect size (*F*(1, 14) = 10.340, *p* = 0.006, *η*^2^ = 0.119); thus, active wheelchair use had lower Borg scores under all trials and was considered less exhausting than the passive wheelchair. In summary, use of the active wheelchair resulted in the users expending less energy per meter travelled and at the same time experiencing less fatigue. This may benefit overall wheelchair mobility and possibly reduce health complications.

## 1. Introduction

Manual wheelchairs are used to promote and improve the efficiency, safety, and independence of mobility. However, travelling long distances at fast speeds and uphill and traversing uneven terrain and stairs are highly physically demanding for the user and can expose the user to the risk of injuries and limit mobility. Observations have revealed that the daily distances travelled by wheelchair users were considerably lower compared to individuals who can resort to regular walking [1, 2]. Ultimately, mobility limitations of users of a manual wheelchair may negatively affect social participation, health, and quality of life.

The extent to which the wheelchair design matches the user's characteristics, physical capacity, and context of use is crucial for successful use. Lin and Sprigle [3] found that the distribution of mass had a more significant influence on manoeuverability than the wheelchair mass itself. Several aspects of the wheelchair configuration, such as the rear axle position, have been found to affect manual propulsion and overall mobility [4]. The rear wheels' axle position has been pointed as a key factor to be considered when optimizing manual wheelchair propulsion performance [3, 5, 6]. Additionally, Van Velzen et al. [7] showed the seat height to be a factor influencing cardiorespiratory parameters and mechanical efficiency during submaximal manual wheelchair propulsion, with an optimal seat height found with an elbow angle ranging from 100° to 130° (measured in a standardized sitting posture).

There is a body of evidence demonstrating the effect of tire type and pressure on wheelchair mobility. The study by Sawatzky et al. [8] found that pneumatic tires have less rolling resistance when inflated to at least 50% when compared to solid tires, and the study highlighted the possible negative consequences of the use of solid tires on the users' mobility and health. This is consistent with the study of de Groot et al. [9], who found that the use of solid tires and tire pressure at 25% was associated with higher oxygen consumption when compared to 100%. Accordingly, Sawatzky et al. [8] reported an increase of 25% in energy expenditure associated with the use of tires inflated below 50%. Similarly, an increase in energy expenditure by over 15% with decreasing tire pressure was reported in children propelling manual wheelchairs at self-selected velocity [10]. Corroborating with these findings, in a study with sports wheelchairs, Mason et al. [11] found a reduction in physiological demand with the use of high-pressure tubular tires and concluded that the tire type and pressure are the most relevant aspects to be considered when it comes to the wheel configuration of sports wheelchair. The study of Ott et al. [12] also reported lower rolling resistance with the use of pneumatic tires.

In addition to the evidence on the effect of rear wheel/tire configuration, caster type was also shown to influence wheelchair mobility. The study of Chan et al. [13] showed that caster size is an important factor, with a small caster (4 in) being associated with higher global rolling resistance when compared to 5 in and 6 in casters. Indeed, the study of Zepeda et al. [14] demonstrated a relationship between caster wheel diameters and drag forces in manual wheelchairs, and their findings indicate that the smaller diameters result in a significant increase in the drag forces when the weight distribution is in a way that the user has at least 30% of his/her weight supported by the caster wheels. Also, the authors found that the weight supported by the caster wheels is a more important factor than the caster wheel diameter when it comes to reducing rolling resistance. This is consistent with the findings from the study of Lin and Sprigle [3] who found a decrease in the propulsion efforts with the increased percentage weight on the rear wheels. In addition, the effect of caster design on wheelchair mobility in winter conditions has been reported [15]. Based on the findings reported in the abovementioned studies, there seems to be a direct correlation between wheel/caster configuration and rolling resistance that, ultimately, influences the physiological demand for manual wheelchair propulsion.

Ultimately, optimizing these configurations can help increase propulsion efficiency and consequently improve overall mobility. Manual wheelchair users, which use exclusively hand rim propulsion, must use their upper limbs to perform most of the daily activities. Over time, this may lead to upper limb pain and injuries, precluding individuals from performing physical activities, and this may result in a more sedentary lifestyle [16, 17]. A sedentary lifestyle can lead to cardiovascular and pulmonary health complications, which is a major cause of early death in this group [18]. Therefore, changes in the wheelchair design and configuration could enable wheelchair users of different physical capabilities and characteristics to move with their wheelchairs more comfortably and effectively. Optimizing the efficiency of manual wheelchair mobility may thus benefit the users by reducing the physical demands needed, thereby improving their ability to move independently and satisfactorily both at home and in their community.

When it comes to the metabolic costs of manual wheelchair propulsion, it is typically quantified by the oxygen uptake per unit time (VO_2_, mL min^−1^ consumption), heart rate, and push frequency, while VO_2_ efficiency is typically quantified by oxygen uptake per distance travelled (VO_2_, mL m^−1^ efficiency) [19]. Metabolic cost and VO_2_ efficiency in wheelchair mobility have been addressed in different fields such as sports [20], comparing different groups of users [21, 22] and different propulsion mechanisms [19]. Additionally, the study of Pierret et al. [23] explored cardiorespiratory responses and perceived exertion during manual wheelchair propulsion at increasing cross slopes. It was found that physiological responses (VO_2_, mL kg^−1^ min^−1^) to slopes of 2% were indistinguishable from flat surfaces while slopes of 8% imposed a critical threshold of physiological strain [23]. This information demonstrates how challenging—or even excluding—the outdoor environments can be, as well as highlights the need to explore the wheelchair factors that are associated with optimal mobility efficiency and challenges in daily life.

Despite the relevant contribution from previous studies, there is, to the best of our knowledge, little multifactorial research on how the wheelchair's design influences cardiorespiratory responses, perceived exertion, and mobility performance.

Given these findings, and the fact that the design of a product can influence the users' physiological responses during interactions, this study is aimed at comparing the design and configuration of the most commonly used manual wheelchair models through cardiorespiratory responses, perceived exertion, and mobility performance using two different manual wheelchairs, during mobility tasks. The chosen models have different characteristics in terms of mass, frame designs, and caster wheels' size. Our hypothesis is that performing mobility tasks with an active rigid frame wheelchair is more energy-efficient and less strenuous compared to performing the same tasks with a passive foldable frame wheelchair.

## 2. Materials and Methods

### 2.1. Experimental Design

A within-group 2 × 3 × 2 controlled experiment was chosen with three independent variables and five dependent variables. The independent variables included mobility devices (wheelchairs), with the levels active and passive; conditions with the levels straight line, slalom, and agility motion; and speed level with levels comfortable and fast. The dependent variables included oxygen uptake (VO_2_, mL min^−1^), distance travelled (m), speed (m sec^−1^), VO_2_ efficiency (VO_2_, mL m^−1^), and perceived physical exertion using the Borg scale [24].

### 2.2. Participants

Participants were recruited through online forms and invitations to the University Campus. A sample of 15 persons (9 men and 6 women) voluntarily participated in this study (Table 1). None of the participants had any disabilities or previous experience with wheelchair use and met the following inclusion criteria: older than 18 years and without recent history of upper limb injuries or any other complaint that could affect their ability to manually propel the wheelchairs. The study was approved by the local ethics committee at UNESP, Brazil, and a risk analysis was submitted to Oslo Metropolitan University, Oslo, Norway, and the plan for handling personal information was approved by the Norwegian Center for Research Data. Written informed consent was obtained prior to participation, and the experiments were conducted according to the Helsinki declaration.

### 2.3. Procedure

Participants were instructed to avoid exercise and alcohol 24 hours prior to testing and to abstain from coffee, tea, and tobacco on the day of testing. A portable metabolic analyzer was used to collect expired air in breath-by-breath mode during all wheelchair propulsion trials, which were performed for 5 minutes continuously repeated.

**Figure 1:**
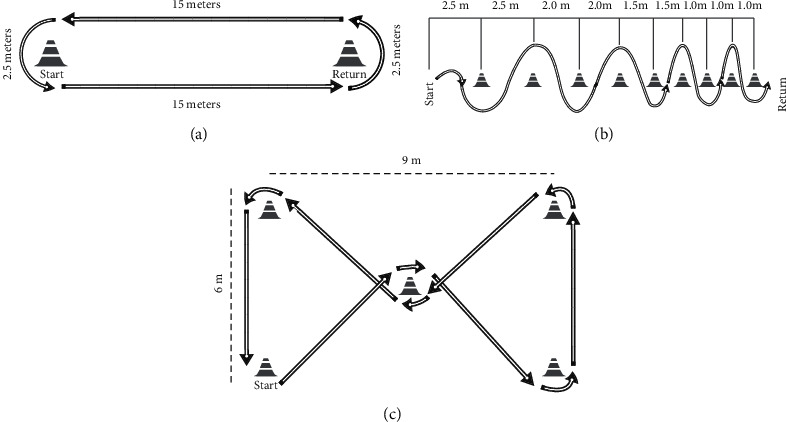
Trajectories: (a) forward; (b) slalom; (c) agility.

The wheelchair propulsion trials consisted of three different conditions (Figure 1) using two different wheelchair models and at two different speed levels, i.e., self-selected pace (comfortable) and as fast as possible (fast). In total, the complete experimental setup consisted of 12 trials. The conditions were forward (15 m straight motion with 2.5 m turning ratio at each end), slalom course (nine cones aligned and separated by decreasing distances, as proposed in the study of Medola et al. [25]), and an agility test with five cones distributed in a rectangular shape (6 meters wide by 9 meters long) with a cone positioned in the center of the rectangle (see Figure 1). The sequence of trajectories, speed levels, and type of wheelchair was randomized to minimize systematic errors and learning effects. The trajectories were performed indoors in a large room of about 150 m^2^. Participants completed all tests without reporting severe fatigue and shoulder or arm pain.

### 2.4. Equipment and Measurements

The portable oxygen analyzer (Metamax 3B; Cortex Biophysik, Leipzig, Germany) was calibrated for barometric pressure and with a reference gas mixture of 16% O_2_ and 4% CO_2_. The calibration was then verified with measurements of ambient air, according to the manufacturer's instructions. Calibration of the flow-volume turbine was performed with a 3 L syringe (Hans Rudolph, Kansas, USA).

Oxygen consumption (VO_2_, mL min^−1^), lung ventilation (VE, L min^−1^), heart rate (HR, beats min^−1^), and respiratory exchange ratio (RER) values were continuously monitored by the oxygen analyzer during testing by real-time telemetry in order to verify steady-state conditions during trials and fatigue effect during resting periods. Immediately prior to and following the completion of a propulsion trial, the participants' responses to a Perceived Exertion Questionnaire were recorded (Borg, 1982). Distance travelled was collected during each trial by a Cateye cycle computer VELO 8 (CATEYE Co., Osaka, Japan) mounted on the left rear wheel of both wheelchairs. The average speed, measured in meters per second, was computed by dividing the distance travelled in each trial situation by the measured total time for each trial situation. In order to measure oxygen efficiency (VO_2_, mL m^−1^), the oxygen consumption (VO_2_, mL min^−1^) was divided by the distance travelled (m) during each trial.

**Figure 2:**
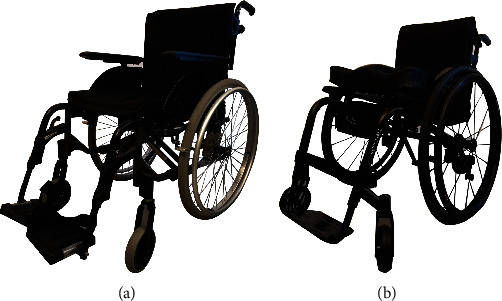
Wheelchairs: (a) passive model; (b) active model.

The wheelchairs used were an active model (Kuschall, K-series, INVACARE—Witterswil, Switzerland) and a passive model (Rea Spirea 4 NG, INVACARE—Witterswil, Switzerland) and presented in Figure 2, as described in the Technical Regulation—TEK 10 (Kartlegging av rullestoler og rullatorer i forbindelse med revisjon av Byggteknisk forskrift–TEK10 [26]). According to TEK 10, active model wheelchairs are destined for users which are mostly active and independent persons, and all-round models are made for users who can either drive themselves or be driven by others. This chair is often used by the elderly or people with disabilities and in need of wheelchair transport over longer distances. There may also be users who for various reasons need more support than an active wheelchair provides, and this all-round model is the most common type of wheelchair on the market.

The active wheelchair had a rigid frame, no armrest but adjustable axle position, tilt angle, and foot support height. The all-round wheelchair with a foldable frame had armrests, adjustable axle position, and backrest angle. A detailed description of measurements of the two wheelchairs is presented in Table 1, demonstrating differences in important aspects such as mass, length, and caster size. Such differences do not allow for addressing specific design factors of the two investigated chairs. However, the research reported here was designed to compare two of the most commonly used wheelchair types.

### 2.5. Propulsion Trials

After reporting to the laboratory, participants had a familiarization period of about 10 minutes or more. During this period, the participants were instructed on how to use both wheelchairs, since they had no previous experience with any wheelchair. Next, they were fitted with the portable oxygen analyzer and subsequently rested for five minutes sitting in the assigned wheelchair. After the rest, the participants commenced performing the different trials in a randomized order. Each trial lasted a total of five minutes, and following the completion of a trial, the participants then rested in the wheelchair for five minutes to decrease the effects of fatigue before commencing the next trial. This procedure was repeated until all 12 trials were completed.

### 2.6. Data Analysis

The independent variables included mobility devices (wheelchairs), with the levels active and passive; conditions with the levels straight line, slalom, and agility motion; and speed level with levels comfortable and fast. The dependent variables included oxygen uptake (VO_2_, mL min^−1^), distance travelled (m), speed (m sec^−1^), VO_2_ efficiency (VO_2_, mL m^−1^), and perceived physical exertion using the Borg scale [24]. Median values with 95% confidence intervals (95% CI) of VO_2_ (mL min^−1^) were analyzed for the last two minutes of each five-minute trial, as previous studies have shown that a steady state is achieved after three minutes of continuous exertion, and measures of metabolic cost and efficiency can be reliably analyzed after three minutes [27]. Median (95% CI) distance, speed, and perceived exertion were also calculated for each trial to verify statistical differences between conditions (comfortable speed and fast speed), wheelchairs (active and passive), and conditions (forward, slalom, and agility).

The data were checked for normality using Shapiro-Wilks tests which showed that none of the datasets were completely normally distributed; hence, the nonparametric Aligned Rank Transform (ART) procedure [28] was used to convert each dataset into aligned ranks which then were analyzed using a traditional three-factor repeated measures ANOVA. In the cases where the assumption of sphericity was not satisfied, a Greenhouse-Geisser correction was applied. For the VO_2_ efficiency analysis, a Wilcoxon test was performed since conducting pairwise comparisons involving multiple factors is not recommended during the ART procedure [28]. Analyses were performed using JASP version 0.11.0.0. Point estimates are therefore presented using medians and spreads using 95% confidence intervals of these medians.

## 3. Results

### 3.1. Participants

A sample of 15 persons (9 men and 6 women), with a mean (SD) age, height, and weight of 31.8 (6.0) years, 174.6 (7.7) cm, and 70.3 (12.1) kg, voluntarily participated in this study; Table 2 summarizes the participants' demographics.

### 3.2. Oxygen Consumption

**Figure 3:**
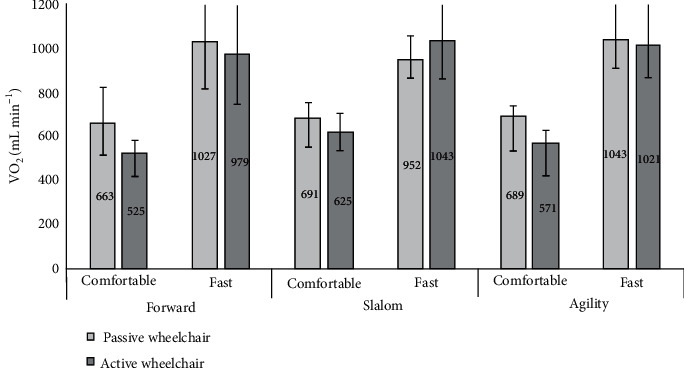
Median oxygen uptake (VO_2_, mL min^−1^). Error bars show 95% confidence intervals of the median.

Figure 3 shows the results of the oxygen consumption measurements under the twelve trials. The oxygen consumption (medians and 95% CI) was the lowest with the active wheelchair in forward motion with comfortable speed (525 (499–809) mL min^−1^) and highest with the same type of wheelchair in the slalom condition with fast speed (1043 (789–1221) mL min^−1^). As shown in Figure 3, the median oxygen consumption is higher under all conditions with the passive wheelchair, except for the slalom condition with fast speed where the active wheelchair median oxygen consumption is higher. However, the 95% confidence intervals overlap to a high extent, and there was not a significant effect of wheelchair type on oxygen consumption (*F*(1, 14) = 3.703, *p* = 0.075).

Figure 3 shows that the results for the three conditions are quite similar, and no significant effect of condition could be detected (*F*(2, 28) = 305.867, *p* = 0.489).

However, the oxygen consumption under the fast speed was much higher than at the comfortable speed, and this difference was significantly different (*F*(1, 14) = 74.822, *p* < 0.001, *η*^2^ = 0.438). The large effect size also confirms that these are statistical differences. Our analysis also revealed an interaction between speed and condition (*F*(2, 28) = 9.665, *p* < 0.001, *η*^2^ = 0.010), but the effect size is negligible.

We also observed an interaction between wheelchair type and speed (*F*(1, 14) = 7.892, *p* = 0.014, *η*^2^ = 0.008), again with a negligible effect size. No interaction effects were found between condition and wheelchair (*F*(2, 28) = 528.317, *p* = 0.058) or between condition, speed, and wheelchair (*F*(2, 28) = 0.468, *p* = 0.631).

### 3.3. Distance

**Figure 4:**
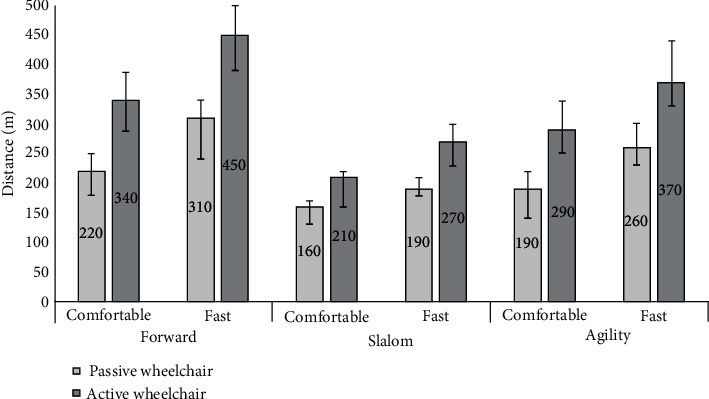
Median distance travelled in meters. Error bars show 95% confidence intervals of the median.

Figure 4 shows the results of the distance measurements. The results show that the longest distances were achieved with the active wheelchair under all trials, which is on average 42% more distance covered under all conditions and speeds, compared to the passive wheelchair. The effect of the wheelchair type on distance was also statistically significant with a large effect size (*F*(1, 14) = 111.5, *p* < 0.001, *η*^2^ = 0.414). Moreover, Figure 4 shows that the longest distances were achieved under the forward condition and the shortest distance under the slalom condition. The conditions also had a statistically significant effect on distance travelled with a large effect size (*F*(1.434, 20.08) = 181.8, *p* < 0.001, *η*^2^ = 0.459). Also, the speed had a statistically significant effect on distance but with a moderate effect size (*F*(1, 14) = 173.2, *p* < 0.001, *η*^2^ = 0.284) as the fast speed consistently resulted in a longer distance travelled compared to the comfortable speed (see Figure 4).

Next, we observed a significant interaction between wheelchair type and conditions (*F*(2, 28) = 8.194, *p* = 0.002, *η*^2^ = 0.036), between wheelchair type and speed (*F*(1, 14) = 7.238, *p* = 0.018, *η*^2^ = 0.019), and also between condition and speed (*F*(2, 28) = 22.394, *p* < 0.001, *η*^2^ = 0.071), but in all three cases, the effect sizes were negligible. No interaction was found across all the three factors of wheelchair type, condition, and speed (*F*(2, 28) = 0.851, *p* = 0.438).

### 3.4. Speed

**Figure 5:**
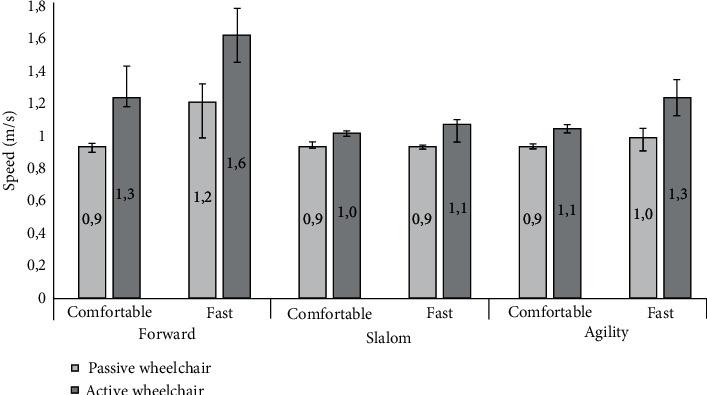
Median speed in m s^−1^. Error bars show 95% confidence intervals of the median.

Figure 5 shows the results of the speed measurements. The results show that the highest speeds were achieved with the active wheelchair under all trials, that is, on average 20% higher speeds with this wheelchair under all conditions and speeds. The effect of the wheelchair type on speed was also statistically significant with a small effect size (*F*(1, 14) = 21.766, *p* < 0.001, *η*^2^ = 0.199). Moreover, Figure 5 shows that the highest speeds were achieved under the forward condition and similar speeds with the slalom and agility conditions. The condition exhibited a statistically significant effect on speed with a negligible effect size (*F*(2, 28) = 0.520, *p* < 0.6, *η*^2^ = 0.004).

As expected, the speed level had a statistically significant effect on the measured speed with a medium effect size (*F*(1, 14) = 17.123, *p* < 0.001, *η*^2^ = 0.063) as Figure 5 shows that the fast speed consistently resulted in a longer distance travelled compared to the comfortable speed.

Next, we observed a nonsignificant interaction between wheelchair type and condition (*F*(2, 28) = 4550.41, *p* < 0.091), between wheelchair and speed (*F*(1, 14) = 3864.2, *p* < 0.204), and also between condition and speed (*F*(2, 28) = 905.150, *p* < 0.600, *η*^2^ = 0.004) and across all the three factors of wheelchair type, condition, and speed (*F*(2, 28) = 309.339, *p* = 0.769, *η*^2^ = 0.001).

### 3.5. Perceived Exertion (Borg Scale 6-20)

**Figure 6:**
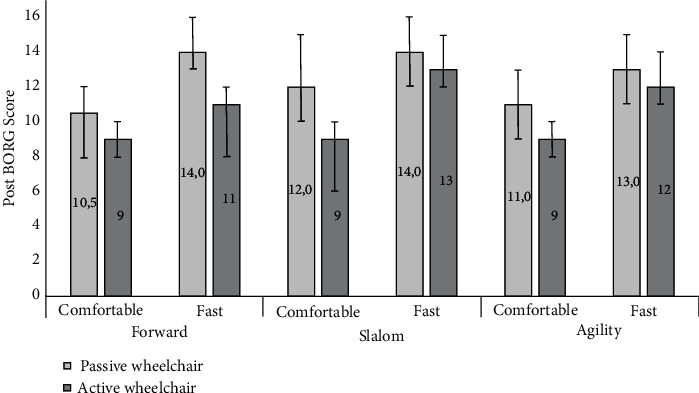
Median post-Borg scores (scale 6-20). Error bars show 95% confidence intervals of the median.

Figure 6 shows the results of the rating of perceived exertion. The results show that the highest Borg scores were observed with the passive wheelchair under all trials; that is, use of the active wheelchair was considered less exhausting. The effect of wheelchair type on Borg scores was also statistically significant but with a small effect size (*F*(1, 14) = 10.340, *p* = 0.006, *η*^2^ = 0.119). Figure 6 does not reveal any obvious difference between the BORG scores for the three conditions. This is confirmed by the statistical test that did not flag any significance (*F*(1.336, 18.707) = 3.924, *p* = 0.052). Speed, on the other hand, did have a statistically significant effect on Borg scores with a moderate effect size (*F*(1, 14) = 69.209, *p* < 0.001, *η*^2^ = 0.264). Figure 4 shows that the fast speed consistently resulted in a higher Borg score compared to the comfortable speed.

No interactions were found between wheelchair and conditions (*F*(1, 14) = 0.82, *p* = 0.78), wheelchair type and speed (*F*(1, 14) = 0.05, *p* = 0.82), condition and speed (*F*(2, 28) = 0.038, *p* < 0.96), and across all the three factors (*F*(2, 28) = 1.627, *p* = 0.214).

### 3.6. Efficiency

**Figure 7:**
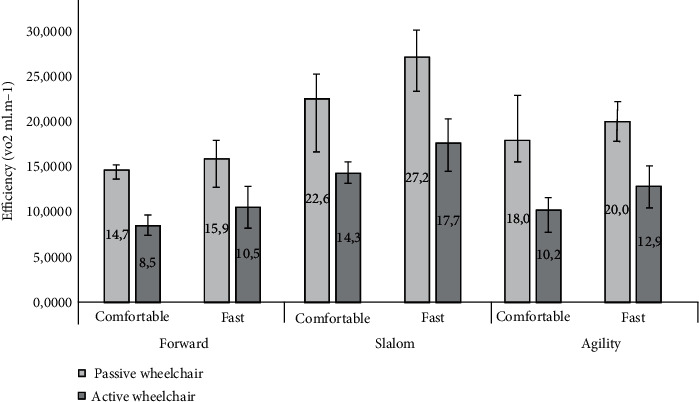
Median efficiency scores (VO_2_, mL m^−1^). Error bars show 95% confidence intervals of the median.

Figure 7 shows the results of the VO_2_ efficiency (VO_2_, mL m^−1^). The results show that the lowest rates of oxygen consumption per meter travelled (highest VO_2_ efficiency) were achieved with the active wheelchair under all trials. The effect of wheelchair type on VO_2_ efficiency was statistically significant with a large effect size (*F*(1, 14) = 118.298, *p* < 0.001, *η*^2^ = 0.541). Moreover, Figure 7 shows that the lowest rates were achieved under the forward condition and the highest rates (lowest efficiency) under the slalom condition. The effect of condition on VO_2_ efficiency was statistically significant with a large effect size (*F*(2, 28) = 203.566, *p* < 0.001, *η*^2^ = 0.508). Also, the speed had a statistically significant effect on VO_2_ efficiency but only with a small effect size (*F*(1, 14) = 16.872, *p* < 0.001, *η*^2^ = 0.139), i.e., the fast speed consistently resulted in a lower rate compared to the comfortable speed.

Next, we observed a significant interaction between wheelchair and condition (*F*(2, 28) = 10.860, *p* = 0.001, *η*^2^ = 0.053) and also between condition and speed (*F*(2, 28) = 6.170, *p* = 0.006, *η*^2^ = 0.020), but in both cases, the effect sizes were negligible. No interaction was found between wheelchair type and speed (*F*(1, 14) = 0.019, *p* = 0.893) or across all the three factors of wheelchair type, condition, and speed (*F*(2, 28) = 0.229, *p* = 0.797).

When comparing wheelchairs in the same trial conditions, e.g., passive with active wheelchair and forward condition at fast speed, the Wilcoxon tests revealed significant differences (*p* < 0.001). These results revealed that an active wheelchair was more VO_2_-efficient, especially during the condition slalom at comfortable speeds, showing 43% less oxygen consumption per meter travelled compared to the passive wheelchair.

## 4. Discussion

The results confirm that the wheelchair design influences the performance of manual wheelchair mobility in terms of cardiorespiratory responses, perceived exertion, and average speed. An active wheelchair with a rigid frame exhibited more beneficial characteristics than the passive wheelchair with a foldable frame as it resulted in better VO_2_ efficiency, higher speeds, and longer distances travelled, and it was perceived as less tiring to move.

The active wheelchair also resulted in lower oxygen consumption under most trials, but not to a level of statistical significance. However, the active wheelchair showed a significantly better VO_2_ efficiency compared with the passive wheelchair in all trials tested. These results are consistent with those of Beekman et al. [29] who found similar efficiency results, with paraplegic participants on a circular track, comparing a low mass rigid frame wheelchair (active model) with a higher mass foldable-frame wheelchair (passive model). Likewise, Hilbers and White [30] also found better efficiency of a sports wheelchair compared with a conventional wheelchair observed at four different speeds.

One important difference between the wheelchairs we tested was the mass (10.3 kg for the active wheelchair and 16 kg for the all-round wheelchair), i.e., a difference of about 55%. Studies have been addressing mass as a potential factor that could influence wheelchair performance and especially physiological (VO_2_ consumption) and perceptual (perceived exertion) outcomes. Sagawa et al. [31] added masses of 1, 2, and 5 kg in a multisport manual wheelchair during six different overground mobility tasks, and for all tests, they did not find a significant effect of mass on VO_2_ consumption, performance, or perceived exertion. Similarly, de Groot et al. [9] found that increased mass did not lead to an increasing physical strain; however, the combination of increased mass and tire type had an effect on the physical strain. In addition to the mass, the wheelchair length and width were also factors of difference between the chairs. The active chair of this study was also more compact (smaller length and width), which influences the system's weight distribution, although we did not measure this in this study. Mass and weight distribution are factors that influence characteristics of the mechanical performance of wheelchairs, and interpreting the current results with the support of these parameters would have provided a more complete view of the relation between wheelchair types, mechanical parameters, and physiological and perceptual outcomes. Mass distribution seems to have a stronger effect on the propulsion efforts more than the general mass [3, 32, 33]. This is in agreement with our observations, namely, that the users adapt to the device, situations, and tasks, and the design of the device as a whole impacts the general mobility of the wheelchair.

The caster wheels' size was also a factor of difference between the wheelchairs in this study, with the active chair having a smaller diameter (4^″^) compared to the passive chair (6.5^″^). Smaller diameters of casters have been associated with an increase in rolling resistance [13, 14]. However, the influence of the caster wheels on rolling resistance seems to be dependent on the weight distribution [3, 14, 34], that is, the percentage of the weight on the drive wheels and on the casters. However, as we did not measure weight distribution, it is not possible to discuss the influence of this factor—caster wheel's size—in the current findings.

Regardless of the lack of a significant difference between the active wheelchair and the passive wheelchair, in terms of oxygen consumption (VO_2_, mL min^−1^), there was a statistical difference in the energy efficiency (VO_2_, mL m^−1^) between the two types of wheelchairs during all performed trials, with the lowest rates (better efficiency) achieved with the active wheelchair.

One explanation for these results may be that the larger mass, wider wheelbase, and more torsion of the chassis of the passive wheelchair with a foldable frame may have resulted in lower cornering speed, especially during the slalom and agility trajectory. Consequently, this results in a lower general speed and shorter distance covered during the five minutes of the task with the foldable wheelchair. This notion is supported by Medola et al. [25], who showed that in a slalom course, more demanding turns resulted in slower speeds, a decrease in the translational component, and an increase in the turning component of the kinetic energy. Thus, it requires repeated braking and accelerating of each wheel in a specific pattern to speed up or slow down the left and the right wheels in order to follow the turns of a slalom course. Our assumption is that the passive wheelchair with its larger mass and wider axles reduces the manoeuverability and, consequently, the speed in a course with many turns. Thus, when oxygen consumption (VO_2_, mL min^−1^) is similar for the two wheelchairs, but speed is different, energy efficiency (VO_2_, mL m^−1^) will be worse (higher rates) for the slower type wheelchair.

The observed effects of condition (forward, slalom, and agility) on distance and speed were as expected, as it is logical to cover longer distances at higher speeds along straight paths than along paths with many turns. In order to navigate around a course with paths, the wheelchair user needs to slow down and hence will cover shorter distances. In terms of distance travelled, no matter the wheelchair type, forward motion had the longest distance travelled and the slalom condition the shortest for both speed levels (comfortable and fast). When looking at the gain in the distance travelled comparing comfortable and fast propulsion, the slalom course had the smallest gain for both passive (18.75%) and active (28.57%) wheelchairs, while the forward trajectory showed the greatest gains (40.90% and 32.35% for the passive and active chairs, respectively). Moving on the agility condition as fast as possible resulted in increases of 36.84% (passive) and 27.58% (active chair) of the distance compared to the comfortable mode.

Such differences can be explained by the characteristics of the trajectories: the slalom course demands the user to manoeuver the chair in progressively tighter spaces (smaller turning radii), which affects the ability to accelerate the chair in a continuous mode, and as a result, the gain in the distance travelled is lower. As shown in the study of Medola et al. [25], the control to manoeuver the chair around the cones comprises actions of pushing and braking the handrims in a specific way to accelerate, brake, and turn the chair. Such coordinated actions are specially challenging to perform under a fast propulsion mode, as there is a progressive decrease in the velocity as the chair moves around the cones positioned closer together [25], and to increase velocity, propulsion costs [35] and torque required to accelerate the chair [31] are higher than to maintain motion. On the other hand, the agility test is composed of curves separated by straight sections that are long enough to allow acceleration recovery and preparation for the manoeuver around the cones, and the gain in distance travelled was closer to the distances observed in the forward condition.

A significant interaction was also observed between condition and speed. An inspection of the data shows that the difference in speed only could be observed in the condition where the wheelchair followed a straight path, while in the conditions with turns, there was no observable difference in speed. This probably occurs due to the complex manoeuvers (slalom and agility) that prevented the participants from achieving the same high speed as during the straight line course.

During comfortable speed and straightforward propulsion, the median (95% CI) rating of perceived exertion (Borg scale) was for the passive wheelchair 10.5 (9-13) and for the active wheelchair 9 (8-10). Similarly, Qi et al. [36] showed results for arm Borg scale ratings during comfortable speed as 9.3, during forward propulsion at comfortable speed with spinal cord injured persons, hence similar to the present findings. However, it should be noted that the perceived exertion during forward propulsion at comfortable speed actually is 30-35% higher than normal resting levels (Borg scale = 6). Hence, even lesser demanding mobility tasks can over time, potentially, fatigue wheelchair users. When it comes to the real user of wheelchairs, long-term overload in the upper limbs may lead to pain and injuries, precluding individuals from performing physical activities, and ultimately, this may result in a more sedentary lifestyle leading to cardiovascular and pulmonary health complications and less social participation [16–18].

In addition, it is interesting that the reported Borg ratings do not increase as the complexity of the task increases. In other words, straight line propulsion compared with mixed trajectories (slalom or agility) does not necessarily result in higher perceived exertion. One reason for this may be that the two latter movements were performed at considerably lower speeds compared to straight line propulsion. However, as expected, the participants reported significantly higher levels of exertion at high speeds compared with comfortable speeds.

The current study has identified that different types of wheelchairs impact cardiorespiratory and perceived exertion, speed, and distance travelled, during various mobility tasks. This ultimately resulted in significant differences in energy efficiency and mechanical responses between the two types of wheelchairs. The active wheelchair with a rigid frame showed to be more VO_2_-efficient when compared with the passive wheelchair with a foldable frame. A better understanding of the physiological parameters of the user and the mechanical aspects of the wheelchair can contribute to an improved understanding of the user/wheelchair system and, as a result, improve general mobility and user satisfaction. Ultimately, this study demonstrates how metabolic responses provide important data on how the design of the wheelchair influences the user and the user's general performance. Furthermore, the active wheelchair results in better VO_2_ economy and less exertion than the passive chair. This makes it possible to travel faster and longer with less fatigue with the active chair.

Although the current study produced important findings, it has limitations that must be noted. One noteworthy limitation of this study is that only participants without disabilities were recruited for this study. The results may therefore not be generalized to expert wheelchair users, as shown in other assistive technology studies involving nondisabled participants (see, for instance, dos Santos et al. [37]). In addition, the mobility tests were all carried out on a flat surface in a controlled environment, which is not representative of the diversity of real-life situations experienced by expert wheelchair users.

The present study compared two different types of wheelchairs that are among the most commonly used models, evaluating them in their conventional configurations, that is, the way they are commercially available. However, since the two devices present differences in important aspects of mobility performance, such as mass, frame design, length, and caster wheels, the results are limited to the comparison of two different types/models of wheelchairs, thus not allowing the evaluation of the effect of specific factors of the wheelchair design and configuration. Thus, although the differences in performance and VO_2_ efficiency between the two wheelchairs resulted from the combination of these different design and configuration factors, it is not possible to identify the impact that each factor had on the findings reported here. Additionally, the wheelchairs were not set to an optimal configuration for each person during the tests, but the configuration of the chairs was left in a standard posture. This decision was influenced by the fact that the different wheelchairs were not equally customizable; hence, the configuration was left standard for both wheelchairs. The inability to configure the wheelchairs optimally for each participant during the tests may have affected the results negatively.

## 5. Conclusions

Solving mobility issues optimally is one of the main challenges of today's wheelchair design problems. The results presented by this study demonstrate how design can influence both the physiological responses of the users and the mechanical measurements (speed and distance travelled) during free movements. Measurements of oxygen consumption provide precise data on propulsion costs during manual wheelchair manoeuvering. When comparing the same trajectory and speed condition, the active wheelchair with a rigid frame obtained better results, for all mechanical and metabolic analyzed variables, although only measurements of the VO_2_ efficiency showed statistical differences and speed level, however with a small effect size. On the other hand, Borg values did not demonstrate significant differences. Basics and simple differences (such as mass, casters' wheel size, and length) between the design and configuration of the wheelchairs can lead to improvement in the performance of the user/wheelchair system. Wheelchair design optimizations are needed to reduce metabolic costs that must be addressed to benefit mobility efficiency and minimize health complications. Improving the wheelchair design can be the quickest way to positively impact a wheelchair user's life. Designers, manufacturers, and health professionals should be aware of the differences between the two types of wheelchairs in the design, prescription, and provisioning of manual wheelchairs.

## Figures and Tables

**Table 1:** Wheelchair descriptions.

Factor	Passive wheelchair	Active wheelchair
Mass	16 kg	10.3 kg
Total length	990 mm	790 mm
Total width	630 mm	590 mm
Seat depth	450 mm	450 mm
Seat width	430 mm	420 mm
Seat height	460 mm	Front: 450 mm Rear: 420 mm
Rear wheels' size	24^″^	24^″^
Axle position (relative position of the rear axle to the front of the wheelchair)	685 mm	485 mm
Rear wheels' tire pressure (100%)	72 psi	145 psi
Caster wheels' size	6.5^″^	4^″^
Rear wheel tire type	Pneumatic	Pneumatic
Caster wheel tire type	Solid	Solid

**Table 2:** Demographics.

Id	Age	Height	Weight	Gender
1	26	174	53	F
2	31	177	71	M
3	29	160	51	F
4	31	186	80	M
5	29	174	74	M
6	42	163	70	M
7	42	167	60	F
8	42	187	81	M
9	25	186	78	F
10	27	176	69	F
11	29	170	98	F
12	28	174	58	M
13	37	175	74	M
14	25	170	59	M
15	34	180	78	M
Average (SD)	31.8 (6.0)	174.6 (7.7)	70.3 (12.1)	

## Data Availability

The dataset is available on request from the first author.
